# Dermatological manifestations common in hospitalized HIV patients

**DOI:** 10.1186/1471-2334-14-S7-P46

**Published:** 2014-10-15

**Authors:** Anca Raducan, Sorin Rugină

**Affiliations:** 1 Second Dermatology Clinic, Colentina Clinical Hospital, Bucharest, Romania; 2 Clinical Infectious Diseases Hospital of Constanța, Romania; 3 Faculty of Medicine, “Ovidius” University, Constanța, Romania

## Background

The aim of the study was to establish the most common dermatological manifestations in HIV hospitalized patients and to correlate the status of cutaneous diseases in relation with the CD4 cell count.

## Methods

During 01.12.2013-28.02.2014, 134 HIV-positive subjects admitted to the hospital were enrolled, registering demographic data (age, gender, socioeconomic and educational status), transmission route, WHO clinical stage of HIV, laboratory parameters (CD4 count, viral load) and HIV treatment regimen. All patients were screened for cutaneous diseases by a dermatologist.

## Results

Out of total of 134 HIV-positive patients, 41 patients had cutaneous manifestations, 28 were male (68.29%) and 13 female (31.70%). Most patients were in the age group 16-30 years (58.53%). The majority of patients belonged to the urban area (65.85%) and had a low educational and socioeconomic status (only 56.09% patients graduated secondary school; 82.92% of the patients were unemployed).

The prevailing route of transmission was parenteral (41.46%), most patients had stage C3 HIV disease (56.09%), and only 24.39% of patients had undetectable viral load. Most patients were undergoing ARV regimen (80.48%), 12.19% were naive and 7.31% had ceased therapy. 17 patients had 2 concomitant dermatological manifestations (41.46%), while 13 were treated for only one skin disease (31.70%); 19.51% patients were diagnosed with 3 concurrent dermatological diseases and 7.31% with 4 simultaneous skin disorders. The most common HIV-related dermatological manifestations were oral candidiasis (78.04%), seborrheic dermatitis (14.63%), lipodystrophy (12.19%), tinea corporis (7.31%), herpes zoster (4.87%), syphilis (4.87%) and cellulitis (4.87%). CD4 counts ranged between 2-1551 cells/µL, with a mean CD4 of 139 cells/µL. Oral candidiasis was correlated with a mean CD4 count of 305 cells/µL, seborrheic dermatitis was related to a mean CD4 of 233 cells/µL, while lipodystrophy was associated with a mean CD4 of 202 cells/µL.

## Conclusion

This study indicates a high prevalence of dermatological manifestations in hospitalized HIV patients, occurring more frequently with the progression of HIV to stage C3-WHO and the decline of the immune function (CD4 <500 cells/µL). Oral candidiasis and seborrheic dermatitis are more common in HIV patients (78.04%, and 14.63% respectively) than in the general population (10-30% and 3-5% respectively). Lipodystrophy is a frequent side effect of long-term ARV treatment and/or a result of severe HIV infection. Therefore, early diagnosis of skin diseases may enhance the quality of life of HIV patients.

